# Detection of Pelvic Inflammatory Disease: Development of an Automated Case-Finding Algorithm Using Administrative Data

**DOI:** 10.1155/2011/428351

**Published:** 2011-11-14

**Authors:** Catherine L. Satterwhite, Onchee Yu, Marsha A. Raebel, Stuart Berman, Penelope P. Howards, Hillard Weinstock, David Kleinbaum, Delia Scholes

**Affiliations:** ^1^Division of STD Prevention, Centers for Disease Control and Prevention, 1600 Clifton Road, Mailstop E-02, Atlanta, GA 30333, USA; ^2^Group Health Research Institute, Group Health Cooperative, Seattle, WA 98101, USA; ^3^Institute for Health Research, Kaiser Permanente, Denver, CO 80231, USA; ^4^Department of Epidemiology, Emory University, Atlanta, GA 30322, USA

## Abstract

ICD-9 codes are conventionally used to identify pelvic inflammatory disease (PID) from administrative data for surveillance purposes. This approach may include non-PID cases. To refine PID case identification among women with ICD-9 codes suggestive of PID, a case-finding algorithm was developed using additional variables. Potential PID cases were identified among women aged 15–44 years at Group Health (GH) and Kaiser Permanente Colorado (KPCO) and verified by medical record review. A classification and regression tree analysis was used to develop the algorithm at GH; validation occurred at KPCO. The positive predictive value (PPV) for using ICD-9 codes alone to identify clinical PID cases was 79%. The algorithm identified PID appropriate treatment and age 15–25 years as predictors. Algorithm sensitivity (GH = 96.4%; KPCO = 90.3%) and PPV (GH = 86.9%; KPCO = 84.5%) were high, but specificity was poor (GH = 45.9%; KPCO = 37.0%). In GH, the algorithm offered a practical alternative to medical record review to further improve PID case identification.

## 1. Introduction

An estimated 770,000 cases of pelvic inflammatory disease (PID) are diagnosed annually in the United States [1]. PID comprises infection and inflammation of the uterus, fallopian tubes, ovaries, and other adjacent tissue and has multiple infectious etiologies, many of which have been demonstrated to be sexually transmitted, including *Chlamydia trachomatis* [2]. *C. trachomatis* has been isolated in approximately one-quarter of patients with a symptomatic PID diagnosis [3].

PID of any etiology may lead to further adverse outcomes, including tubal-factor infertility, ectopic pregnancy, and chronic pelvic pain [2]; about 10%–20% of PID cases are associated with infertility and ectopic pregnancy [3]. The specific contribution of chlamydia and other infections associated with PID (e.g., *Neisseria gonorrhoeae* and *Mycoplasma genitalium*) to each of these adverse outcomes is unknown [4, 5]. However, among infertile couples using assisted reproductive therapy, 10–20% are diagnosed with tubal infertility [6, 7]. In an effort to prevent PID and subsequent infertility, chlamydia screening is recommended for all sexually active women aged <25 years [8, 9]. Prior studies have suggested that screening can reduce the risk of PID development by up to 50% [10, 11]. 

While monitoring trends in PID is a critical component to quantifying the public health burden of PID and evaluating the impact of chlamydia prevention efforts, PID surveillance is challenging. In the absence of a laboratory-based case definition, PID is diagnosed on the basis of clinical signs and symptoms [12]. The Centers for Disease Control and Prevention (CDC) recommends empiric treatment for PID when young women have lower abdominal pain with no other clear cause, accompanied by either uterine or adnexal or cervical motion tenderness [8]. Thus, the clinical diagnosis lacks specificity. The “gold standard” for diagnosing tubal infection is laparoscopy, an invasive procedure that is rarely performed in clinical practice [13]. To identify PID cases for research and surveillance purposes, medical record review provides the best method of verifying a clinical diagnosis of PID. In lieu of medical record reviews, ICD-9 codes have been used to identify PID cases from administrative data. A clinical diagnosis of PID may be represented by several ICD-9 codes. The most commonly referenced ICD-9 code, 614.9 (female pelvic inflammatory disease not otherwise specified) has a positive predictive value (PPV) of only 18.1% for the PID surveillance case definition, a substantially stricter definition than the clinical definition used for empiric treatment [14]. When coupled with a positive chlamydia test, the PPV increases to 56%; however, laboratory test results are frequently unavailable in the administrative datasets used to examine PID rates and trends. Information is lacking on the PPV of the multiple ICD-9 codes currently in use for surveillance of acute PID.

Potential PID cases identified from administrative data using ICD-9 codes include some women without PID. To further refine identification of clinically diagnosed PID in the subset of women with an ICD-9 code suggestive of PID, medical record review is preferable but is costly. Applying a PID case-finding algorithm based on additional administrative data to potential PID cases identified from ICD-9 codes may be more practical and allow for more accurate burden and trend ascertainment for PID surveillance activities. The purpose of this analysis was twofold: (1) to determine the PPV of using ICD-9 codes alone to identify PID and (2) to develop a PID case-finding algorithm using administrative data elements to easily identify PID cases among women with PID-related ICD-9 diagnostic codes.

## 2. Materials and Methods

Data from two mixed model healthcare organizations, Group Health Cooperative (GH, Seattle, Wash, USA) and Kaiser Permanente Colorado (KPCO, Denver, CO), were used in this analysis. In 2006, approximately 125,000 women between the ages of 15 and 44 years were enrolled in GH, and about 116,000 women of the same ages were enrolled in KPCO. Both organizations maintain extensive automated administrative and clinical data including enrollment information, demographics, health care utilization, diagnoses, procedures, laboratory tests, and pharmacy records on each enrollee.

### 2.1. Data Collection

A set of 14 ICD-9 codes used in other epidemiologic evaluations of PID was used to identify women with potential PID cases in the GH administrative database (Table 1) [1, 15]. Only codes for PID not identified as chronic were considered, since these cases may be more likely to represent PID cases associated with infectious causes such as chlamydia that could be prevented by screening efforts. PID diagnoses that occurred within 60 days of each other were considered the same PID episode. Using GH data from 2003 to 2007, there were 2,764 total potential PID cases among women aged 15 to 44 years. During this time period, PID rates declined slightly from 568 cases/100,000 person-years to 473/100,000; these data have been described in another publication [16]. From the 2,764 total potential cases, a sample of 393 potential cases was randomly selected for medical record review to determine if the clinical diagnosis was PID. If a woman had multiple PID diagnoses from 2003 to 2007, only the first PID episode was included in the sample. The distribution of ICD-9 codes associated with the 393 potential cases is shown in Table 1; multiple ICD-9 codes could have been selected for the visit associated with each potential PID case. 

Determination of the actual PID case status (i.e., clinical diagnosis) was made by reviewing electronic medical records using a structured chart review instrument. Potential PID cases were confirmed as being clinician-diagnosed cases or not based on explicit clinician documentation of PID used in the context of diagnosis during the visit (e.g., “PID,” “pelvic inflammatory disease,” “pelvic infection,” “salpingitis,” etc.); PID documentation used for patient evaluation (e.g., “rule-out PID”) was not considered. The determination of clinical PID status was made regardless of the clinical signs or symptoms indicated to support such a diagnosis. Cases where the clinical status was uncertain were further reviewed by a study team member (DS). 

In addition to ICD-9 diagnosis codes for PID shown in Table 1, other variables potentially associated with PID were extracted from the GH administrative data to be evaluated as potential predictors in the development of the PID case-finding algorithm. These included age at diagnosis, treatment for PID, inpatient admission, whether chlamydia testing was conducted, and other diagnoses occurring 7 days prior to the first PID diagnosis through 7 days after the last PID diagnosis in an episode. These other possible diagnoses were defined by ICD-9 codes and included appendicitis, ovarian cysts, ectopic pregnancy, pyelonephritis, pancreatitis, leiomyoma, and endometriosis. Treatment appropriate for PID was defined as levofloxacin (500 mg orally once per day for 14 days) or ofloxacin (400 mg orally twice per day for 14 days) based on 2006 recommended PID treatment [12]; other possible antimicrobial regimens for PID treatment were also included. 

Administrative and medical record data from KPCO were used as an external validation dataset to evaluate the performance of the PID case-finding algorithm in another setting. In the KPCO administrative data from 2003 to 2008, 2,685 potential PID cases among women aged 15 to 44 years were identified using the same ICD-9 codes (Table 1). Of these, 500 were randomly selected for medical record review to determine the clinical PID case status. The same structured chart review instrument that was used in the GH development dataset was used for the medical record review at KPCO. All study procedures received human subjects review and approval at each institution (GH and KPCO).

### 2.2. Statistical Analysis

A classification and regression tree (CART) analysis was performed to develop a PID case-finding algorithm using the GH dataset. CART has previously been used to improve ectopic pregnancy case finding and to identify diabetes cases [17, 18]. The algorithm goal in this analysis was to identify additional widely-available variables from administrative data that would aid in predicting clinical PID cases as defined by the medical record review. CART is a nonparametric, binary recursive partitioning method that builds a decision tree or a classification algorithm by splitting data into two groups at each branch (or “node”) [19]. Important predictors are hierarchically identified, and potential cases are classified as PID cases or not at each node. In this analysis, potential predictors considered included ICD-9 codes (Table 1), age at diagnosis, whether treatment appropriate for PID was given, inpatient admission, whether chlamydia testing was conducted, and other concurrent diagnoses (described above). This process is repeated multiple times until the optimal tree is built. At each branch, data are optimally split to maximize the differentiation of observations based on the dependent variable; in this case, the dependent variable was a confirmed clinical PID diagnosis (yes/no) from medical record review. 

The PID case-finding algorithm developed using GH data was then applied to the KPCO data. Algorithm performance was assessed by comparing the PID case status predicted by the algorithm to the PID case status determined by medical record review in each sample dataset (GH and KPCO). Summary statistics evaluating the performance of the algorithm were calculated in the sample population of women with ICD-9 codes related to PID; the medical record review results were assumed to be the truth. Sensitivity was defined as the proportion of PID cases correctly classified by ICD-9 code and confirmed by medical record review as PID cases that were identified as PID by the algorithm. Specificity was the proportion of PID cases identified by ICD-9 code but determined to not be PID that were correctly classified as not PID by the algorithm. Negative predictive value (NPV) was calculated as the proportion of potential cases classified by the algorithm as not PID that were identified by ICD-9 code and found not to be PID by medical record review. Positive predictive value (PPV) was defined as the proportion of algorithm-classified PID cases that were confirmed to be PID by medical record review. The PPV of selecting PID cases using ICD-9 codes alone was also calculated. The overall misclassification proportion was calculated as the proportion of potential PID cases incorrectly classified by the algorithm when compared to the medical record review findings. The 95% confidence intervals (CIs) based on the binomial distribution were calculated for all performance measures. 

Analyses were conducted using SAS version 9.1.2 (SAS Institute Inc., Cary, NC), R (R Foundation for Statistical Computing, Vienna, Austria), and OpenEpi [20, 21]. The CART analysis was performed using “rpart” in the R package. 

## 3. Results

Among the sample of 393 potential PID cases identified at GH using ICD-9 codes alone, 275 (70.0%) were confirmed to be clinical PID based on medical record review; 74 (18.8%) were not PID; 6 (1.5%) were of uncertain case status; 38 (9.7%) had no information available regarding the visit where the PID ICD-9 code was recorded (Table 2). Of the sample of 500 potential KPCO PID cases, 349 (69.8%) were confirmed to be PID, 92 (18.4%) were not PID, 5 (1.0%) were uncertain, and 54 (10.8%) had no information available on the visit. 

Of the 14 ICD-9 codes used to identify potential PID cases from GH, 614.9 was the most common, associated with 64.1% of the 393 potential cases (Table 1). The majority of visits where a potential PID case was identified had only one ICD-9 selected (92.4%), 5.9% had two codes, and 1.8% had three or more. In GH, 68.0% of the 275 confirmed PID cases had an ICD-9 code of 614.9. Of the 500 potential PID cases at KPCO, 50.4% were coded with the 614.9 ICD-9 code; 48.4% of the 441 confirmed PID cases had the 614.9 code recorded. 

When using ICD-9 codes alone to identify PID cases at GH, the PPV was 78.8% (95% CI: 74.1–83.0%). The results were similar for KPCO, where the PPV for using ICD-9 codes was 79.1% (95% CI: 75.0–82.8).

**Figure 1:**
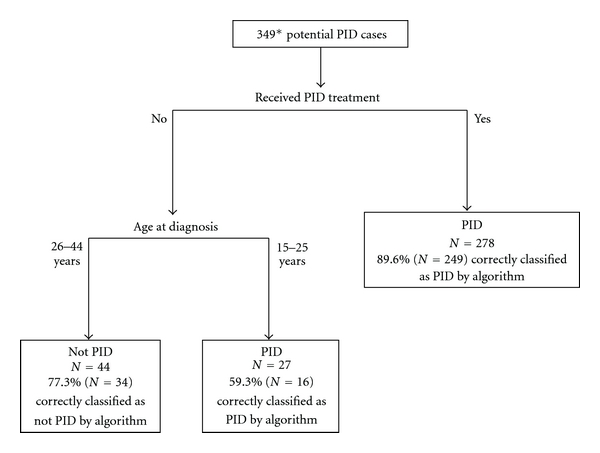
Case-finding algorithm developed using automated administrative data from Group Health Cooperative to refine identification of PID among a sample of women with ICD-9 codes suggestive of PID. PID: pelvic inflammatory disease. *Of 393 potential PID cases with ICD-9 codes associated with PID, 44 were not included due to uncertainty of PID case status after medical record review.

The PID case-finding algorithm is shown in Figure 1. Of the 393 potential PID cases at GH, the 44 with uncertain case status or no information available were excluded. Thus, 349 potential PID cases were used to develop the algorithm. Two predictors of clinical PID were identified by the algorithm. The strongest predictor identified was the presence of treatment appropriate for PID. The algorithm classified 278 potential cases with documented treatment in administrative data as PID cases, of which 249 (89.6%) were confirmed as clinically diagnosed PID. Among those women with no PID treatment recorded, younger age was found to be the most important predictor. Specifically, young women between 15–25 years of age who had not received PID treatment were classified by the algorithm as PID cases. Among 27 such women, 16 (59.3%) were confirmed PID cases. Among 44 women who had no PID treatment and were aged 26–44 years, 34 (77.3%) were correctly classified by the algorithm as not having PID. No specific ICD-9 code was a stronger predictor than PID treatment and age. 

The summary statistics of the algorithm performance using GH and KPCO sample data are shown in Table 3. At GH, algorithm sensitivity was 96.4% (95% CI: 93.4–98.2%), specificity was 45.9% (95% CI: 34.3–57.9%), NPV was 77.3% (95% CI: 62.2–88.5%), and PPV was 86.9% (95% CI: 82.9–90.5%). Using the algorithm, 14.3% of potential PID cases in the sample population of women with ICD-9 codes related to PID were misclassified. When applied to the validation dataset from KPCO, algorithm sensitivity was 90.3% (95% CI: 86.7%–93.2%), specificity was 37.0% (95% CI: 27.1%–47.7%), NPV was 50.0% (95% CI: 37.6%–62.4%), and PPV was 84.5% (95% CI: 80.4–88.0); 20.9% of the potential cases were misclassified.

The distribution of the two predictors included in the algorithm was similar for PID treatment between GH and KPCO potential PID cases, but different for age at PID diagnosis. In GH, 90.6% (249/275) of confirmed PID cases had documented treatment appropriate for PID, compared to 39.2% (29/74) of cases found not to be PID. Likewise, in KPCO, 84.0% (293/349) of confirmed PID cases had documented appropriate antimicrobial treatment, compared to 38.0% (35/92) of non-PID cases. When examining age at diagnosis in GH, 49.1% (135/275) of confirmed PID cases were <26 years, compared to 28.4% (21/74) of cases confirmed by medical record review to not be PID. However, in KPCO women aged <26 years accounted for 38.1% (133/349) of confirmed PID cases and 41.3% (38/92) of cases that were not PID.

## 4. Discussion

One of the primary goals in STD prevention is to reduce the burden of STD-associated infertility. Monitoring trends in PID, an intermediate adverse outcome between STD acquisition and the development of infertility, may help identify progress in STD prevention. However, surveillance of PID, which often relies on case identification from administrative data sources, has been historically difficult. 

To identify clinical diagnoses of PID from medical records data for surveillance and research purposes, administrators and researchers have traditionally relied solely on ICD-9 diagnostic codes. In this study, using a standard set of ICD-9 codes to identify potential PID cases, a simple approach, the PPV relative to medical record review, was fairly high, about 79% at both sites. However, use of ICD-9 codes has limitations, including a lack of specificity [14], nonstandard application (especially when multiple codes may be used to designate a condition), and varying usage across individuals and healthcare sites in selecting which ICD-9 codes to use. 

The algorithm developed in this analysis incorporated additional automated data elements as a practical alternative to medical record review to improve PID case finding among the subset of women with PID-related ICD-9 codes. Algorithm sensitivity (GH = 96.4%; KPCO = 90.3%) and PPV (GH = 86.9%; KPCO = 84.5%) were high at both sites, but higher at the site where the algorithm was developed (GH). However, specificity and NPV were low at both sites, although, again, performance was better at the algorithm development site. In GH, the proportion of potential PID cases misclassified by the algorithm was 14.3%; at KPCO, the proportion of cases that were misclassified using algorithm was 20.9%. Thus, the algorithm developed using GH data did not appear to perform as well in this second site. However, when using ICD-9 codes alone to identify potential PID cases, the PPV was 79%; the PPV increased to 85% in KPCO and 87% in GH when the algorithm was applied. Given the challenges in case identification and PID surveillance, small improvements such as the availability of a case-finding algorithm offer opportunities to move beyond the practice of identifying PID cases based only on ICD-9 codes. As this study indicates, the extent of such value may be specific to the population under evaluation. 

Currently, due to widespread data limitations, public health professionals must rely primarily on ecologic comparisons of STD incidence trends, PID diagnosis trends, and concurrent sexually transmitted disease (STD) prevention activities to evaluate programmatic impact. As data systems improve, ascertainment of STD-specific PID diagnoses may be possible with better automated linkages between laboratory data, clinical data, and inclusion of additional administrative data. The expanded use of electronic medical records will likely offer opportunities to further enhance surveillance of STD-associated PID. The identification of possible methods to improve PID case finding will be a contributing factor.

This analysis has several limitations. Due to budget and time constraints, only a limited number of medical record abstractions were possible and thus, the algorithm developed may not be robust. A larger sample may have resulted in a different algorithm. A random sample of potential PID cases based on ICD-9 diagnostic codes was selected in both sites, and the data used to develop and validate the algorithm should be generally representative of the entire population of potential PID cases with an associated ICD-9 code in GH and KPCO during the study period. In this analysis, potential PID cases were identified using ICD-9 codes; ICD-9 codes may not be applied consistently across healthcare settings. No information on the population with PID not identified by ICD-9 codes (false negatives) was available; therefore, the sensitivity, specificity, and negative predictive value of using ICD-9 codes alone to identify clinically diagnosed PID cases could not be determined. Lastly, the algorithm performance statistics did not include women whose clinical PID status was missing or unable to be determined; fortunately, this finding occurred in a fairly low proportion of potential cases. While the algorithm would classify these cases as PID or not, the performance of the algorithm could not be assessed without medical record review information.

Strengths of this analysis include an evaluation of the group of ICD-9 codes currently in use to identify PID in administrative data. This study is one of the first to examine how these codes perform relative to clinical PID case detection as defined by medical record review. The utilization of CART methodology represents another strength. This strategy allowed for a comprehensive evaluation of additional available automated predictors of clinically diagnosed PID cases and all possible value splits of those predictors without the necessity of making assumptions about underlying variable distributions (a nonparametric approach). Interpretation of the CART findings is straightforward and was easily applied to another external setting (KPCO) after initial algorithm development to allow for an assessment of algorithm robustness. 

## 5. Conclusions

Monitoring PID is important in assessing STD prevention and control efforts, particularly prevention of chlamydia and gonorrhea. The approaches utilized in this analysis may help improve PID surveillance efforts. While the challenge of diagnosing PID remains, results in the two study settings show that the PPV of using ICD-9 codes alone to predict a clinical PID diagnosis was quite high. At GH, the PID case-finding algorithm also offers a practical alternative to further refine PID case identification among the group of women with ICD-9 codes suggestive of PID. Further exploration of case-findings predictors in a larger sample may result in a more robust and generalizable algorithm. Research on additional novel approaches to identify clinical PID cases from administrative data should continue.

## Figures and Tables

**Table 1:** ICD-9 codes commonly utilized to identify possible acute pelvic inflammatory disease (PID) and code distribution among potential PID cases sampled from Group Health Cooperative.

ICD-9 Code and Description	Number of potential cases with code* (%)
098.10-Acute GC upper GU tract, site unspecified	
098.16-Acute GC endometritis	
098.17-Acute GC salpingitis	5 (1.3)
098.19-Acute GC upper GU tract, other site	
098.86-Acute GC peritonitis	

099.56-Acute CT peritonitis	0 (0.0)

614.0-Acute salpingo-oophoritis	
614.5-Acute or unspecified pelvic peritonitis	8 (2.0)
614.8-Other specified inflammatory disease, female pelvic organs	

614.2-Salpingitis/oophoritis, not acute or chronic	22 (5.6)

614.3-Acute parametritis/PID	53 (13.5)

614.9-Unspecified inflammatory disease, female pelvic organs	252 (64.1)

615.0-Inflammatory disease of uterus, except cervix	15 (3.8)

615.9-Unspecified inflammatory disease of uterus	80 (20.4)

GC: gonorrhea, GU: genitourinary, CT: chlamydia.

*A single potential PID case may include multiple ICD-9 codes. 393 total potential PID cases were identified, and a total of 435 ICD-9 codes were used.

**Table 2:** Results from medical record reviews to assess PID cases status at Group Health Cooperative (GH) and Kaiser Permanente Colorado (KPCO).

	PID diagnosis based on medical record review (%)				Total
	PID	Not PID	Uncertain	No information	
GH development dataset	275 (70.0)	74 (18.8)	6 (1.5)	38 (9.7)	393
KPCO validation dataset	349 (69.8)	92 (18.4)	5 (1.0)	54 (10.8)	500

PID: pelvic inflammatory disease.

**Table:** (a) Accuracy of PID case-finding algorithm: GH development dataset.

		New algorithm classification		Total
		Not PID	PID	
Chart-confirmed Diagnosis	Not PID	34	40	74

PID	10	265	275

	Total	44	305	349

**Table:** (b) Accuracy of PID case-finding algorithm: KPCO validation dataset.

		New algorithm classification		Total
		Not PID	PID	

Chart-confirmed Diagnosis	Not PID	34	58	92
	PID	34	315	349

	Total	68	373	441

**Table:** (c)

Performance Statistics (95% CI)	GH development dataset	KPCO validation dataset

PID case identification using ICD-9 codes* alone		
PPV	78.8% (74.1–83.0%)	79.1% (75.0–82.8%)
PID case identification using algorithm		
Sensitivity	96.4% (93.4–98.2%)	90.3% (86.7–93.2%)
Specificity	45.9% (34.3–57.9%)	37.0% (27.1–47.7%)
PPV	86.9% (82.6–90.5%)	84.5% (80.4–88.0%)
NPV	77.3% (62.2–88.5%)	50.0% (37.6–62.4%)
Proportion of potential cases misclassified	14.3% (10.8–18.5%)	20.9% (17.2–25.0%)

*ICD-9 codes shown in Table 1. Only potential cases with complete chart-review information are included.

GH: Group Health, KPCO: Kaiser Permanente Colorado, PID: pelvic inflammatory disease, CI: confidence interval, PPV: positive predictive value, NPV: negative predictive value.

## References

[1] Sutton MY, Sternberg M, Zaidi A, Louis MES, Markowitz LE (2005). Trends in pelvic inflammatory disease hospital discharges and ambulatory visits, United States, 1985–2001. *Sexually Transmitted Diseases*.

[2] Paavonen J, Westrom L, Eschenbach D, Holmes KK, Sparling PF, Stamm WE (2008). Pelvic inflammatory disease. *Sexually Transmitted Diseases*.

[3] Haggerty CL, Gottlieb SL, Taylor BD, Low N, Xu F, Ness RB (2010). Risk of sequelae after *Chlamydia trachomatis* genital infection in women. *Journal of Infectious Diseases*.

[4] Oakeshott P, Kerry S, Aghaizu A (2010). Randomised controlled trial of screening for *Chlamydia trachomatis* to prevent pelvic inflammatory disease: the POPI (prevention of pelvic infection) trial. *BMJ*.

[5] Wallace LA, Scoular A, Hart G, Reid M, Wilson P, Goldberg DJ (2008). What is the excess risk of infertility in women after genital chlamydia infection? A systematic review of the evidence. *Sexually Transmitted Infections*.

[6] Macaluso M, Wright-Schnapp TJ, Chandra A (2010). A public health focus on infertility prevention, detection, and management. *Fertility and Sterility*.

[7] Centers for Disease Control and Prevention (2009). *Assisted Reproductive Therapy Success Rates: National Summary and Fertility Clinic Reports*.

[8] Centers for Disease Control and Prevention (2010). Sexually transmitted diseases treatment guidelines, 2010. *MMWR*.

[9] Calonge N, Petitti DB, DeWitt TG (2007). Screening for chlamydial infection: U.S. preventive services task force recommendation statement. *Annals of Internal Medicine*.

[10] Østergaard L, Andersen B, Møller JK, Olesen F (2000). Home sampling versus conventional swab sampling for screening of *Chlamydia trachomatis* in women: a cluster-randomized 1-year follow-up study. *Clinical Infectious Diseases*.

[11] Scholes D, Stergachis A, Heidrich FE, Andrilla H, Holmes KK, Stamm WE (1996). Prevention of pelvic inflammatory disease by screening for cervical chlamydial infection. *The New England Journal of Medicine*.

[12] Centers for Disease Control and Prevention (2006). Sexually transmitted diseases treatment guidelines, 2006. *MMWR*.

[13] Simms I, Warburton F, Weström L (2003). Diagnosis of pelvic inflammatory disease: time for a rethink. *Sexually Transmitted Infections*.

[14] Ratelle S, Yokoe D, Blejan C (2003). Predictive value of clinical diagnostic codes for the CDC case definition of pelvic inflammatory disease (PID): implications for surveillance. *Sexually Transmitted Diseases*.

[15] Bohm MK, Newman L, Satterwhite CL, Tao G, Weinstock HS (2010). Pelvic inflammatory disease among privately insured women, United States, 2001–2005. *Sexually Transmitted Diseases*.

[16] Scholes D, Satterwhite CL, Yu O Long-term trends in *Chlamydia trachomatis* infections and related outcomes in a U.S. managed care population.

[17] Scholes D, Yu O, Raebel MA, Trabert B, Holt VL (2011). Improving automated case finding for ectopic pregnancy using a classification algorithm. *Human Reproduction*.

[18] Lo-Ciganic W, Zgibor JC, Ruppert K, Arena VC, Stone RA (2011). Identifying type 1 and type 2 diabetic cases using administrative data: a tree-structured model. *Journal of Diabetes Science and Technology*.

[19] Breiman L, Friedman J, Stone CJ (1984). *Classification and Regression Trees*.

[20] Dean A, Sullivan K, Soe M (2010). OpenEpi: open source epidemiologic statistics for public health, version 2.3.1. http://www.openepi.com/OE2.3/Menu/OpenEpiMenu.htm.

[21] RDC Team R: a language and environment for statistical computing. http://www.r-project.org.

